# Semi-Automatic segmentation of multiple mouse embryos in MR images

**DOI:** 10.1186/1471-2105-12-237

**Published:** 2011-06-16

**Authors:** Leila Baghdadi, Mojdeh Zamyadi, John G Sled, Jürgen E Schneider, Shuomo Bhattacharya, R Mark Henkelman, Jason P Lerch

**Affiliations:** 1 Mouse Imaging Centre, The Hospital for Sick Children, Toronto, Canada; 2 Department of Medical Biophysics, University of Toronto, Toronto, Canada; 3 Department of Cardiovascular Medicine, University of Oxford, Oxford, UK

## Abstract

**Background:**

The motivation behind this paper is to aid the automatic phenotyping of mouse embryos, wherein multiple embryos embedded within a single tube were scanned using Magnetic Resonance Imaging (MRI).

**Results:**

Our algorithm, a modified version of the simplex deformable model of Delingette, addresses various issues with deformable models including initialization and inability to adapt to boundary concavities. In addition, it proposes a novel technique for automatic collision detection of multiple objects which are being segmented simultaneously, hence avoiding major leaks into adjacent neighbouring structures. We address the initialization problem by introducing balloon forces which expand the initial spherical models close to the true boundaries of the embryos. This results in models which are less sensitive to initial minimum of two fold after each stage of deformation. To determine collision during segmentation, our unique collision detection algorithm finds the intersection between binary masks created from the deformed models after every few iterations of the deformation and modifies the segmentation parameters accordingly hence avoiding collision.

We have segmented six tubes of three dimensional MR images of multiple mouse embryos using our modified deformable model algorithm. We have then validated the results of the our semi-automatic segmentation versus manual segmentation of the same embryos. Our Validation shows that except paws and tails we have been able to segment the mouse embryos with minor error.

**Conclusions:**

This paper describes our novel multiple object segmentation technique with collision detection using a modified deformable model algorithm. Further, it presents the results of segmenting magnetic resonance images of up to 32 mouse embryos stacked in one gel filled test tube and creating 32 individual masks.

## 1 Background

The mouse, due its genetic similarity to humans, the existence of sophisticated genetic tools to manipulate its genome, rapid reproduction time and comparatively low housing costs, has become an increasingly important model organism in mammalian biology. More recently, Magnetic Resonance Imaging (MRI) has emerged as tool for capturing three-dimensional anatomy to aid in phenotyping mice [1]. There is, however, a resolution to time trade-off in MRI which impacts its application to the mouse, where high resolution is essential due to the small size of the animal but is achieved with very long scan time. Parallelization of MRI experiments is one technique to compensate for the long imaging time. This encompasses either separate transmit/receive coils for each mouse [2], or packing multiple specimen into the same field of view while using a single coil. The latter strategy was chosen by Schneider et al. for phenotyping mouse embryos, placing 32 fixed specimen embedded in gel into a single tube which was then imaged using high-resolution MRI at 9.4 Tesla [3].

**Figure 1:**
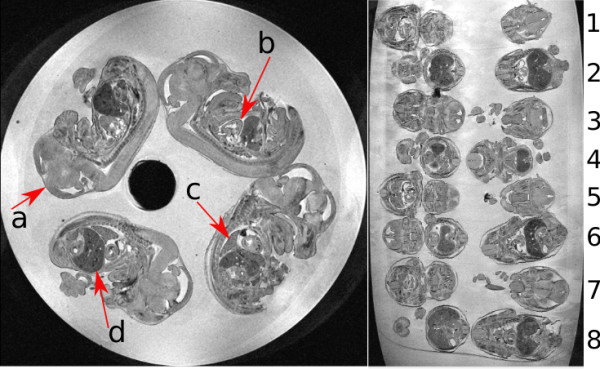
**Two different views of the tube of embryos with arrows showing**. a) brain, b) heart, c) lung and d) liver. Note thirty two embryos in one tube consisting of four embryos in each layer with eight layers.

A complication of the multiple specimens within one field of view strategy, however, is the need to segment the image to isolate the individual specimens. Most available published segmentation algorithms, moreover, fail this task. The two primary reasons are: (1) inhomogeneities of intensity values within the gel and overlap between embryo and gel intensities, ruling out simple thresholding algorithms; and (2) multiple specimens within the imaging tube are touching, ruling out region growing or snake algorithms as they leak into adjacent touching embryos (Figure 1).

Analysis of these datasets thus requires an algorithm which is not only capable of segmenting multiple embryos at the same time but can also separate adjacent touching embryos during segmentation. In this paper, we demonstrate a semi-automatic multiple embryo segmentation technique which allows us to create **individual masks for all embryos **starting from identical spheres. These masks can then be used to separate each embryo from the rest of the embryos in the tube for further analysis. Our method is a modified version of the Simplex Mesh Deformable Algorithm of Delingette [4] with additional balloon and collision forces which allow for segmentation of each embryo as well as its satisfactory separation from neighbouring embryos.

## 2 Methods

### 2.1 Algorithm

#### 2.1.1 Related Work

The deformable simplex mesh algorithm is a general shape reconstruction technique which attempts to build a geometric model from a three-dimensional dataset. Its surface representation is a simplex mesh with topology dual to that of triangulation [5]. In addition, each vertex of a simplex mesh has a fixed number of neighbouring vertices directly connected to it. Because of this geometry, simplex meshes defined discrete concepts at each vertex such as mean curvature and normal vectors which are used for the calculation of forces of deformation. This differs with triangle meshes in that each vertex of a triangle mesh can have many neighbours and concepts such as normal vectors are usually defined for each surface(made of three vertices) of the triangle rather than each vertex. For these reasons, simplex meshes are more preferable as deformable models rather than triangle meshes [6]. On a three-dimensional simplex mesh, each vertex has three neighbouring vertices which define its tangent plane and normal vector. Further, the angle between adjacent edges can be defined as a simplex angle which defines the local shape around each vertex. This angle can then be used for the computation of mean curvature, a measure of surface bending. Finally, the position of a simplex vertex can be defined as a function of its neighbouring vertices and the shape parameters. Due to the large extent of shape control, simplex meshes are better suited for deformation and smoothing than triangulation [5,7].

Just like most other deformable model schemes, each vertex of a simplex mesh is considered as a physical mass subject to equations of motion. To compute the evolution of each vertex *p *of the simplex mesh in a discrete time step *t*, we used the equation of motion(1)

Both forces are calculated at time *t*, **F***_int _*is the internal force which enforces geometric continuity of the mesh whereas **F***_ext _*constrains the distance between the mesh and a three-dimensional dataset.

Internal forces are calculated based on the geometry of simplex meshes and can be decomposed into a tangential force **F***_tangent _*and a normal force **F***_normal_*.

The tangent force controls the position of each vertex with respect to its three neighbours in the tangent plane. The normal force constrains the mean curvature of the surface through the simplex angle of each vertex. For details of how these forces are calculated see [5].

The external force is dependent on the three dimensional dataset and is always directed along the normal (*n*) direction at the vertex *p*. This not only guarantees a smooth deformation of mesh over time but ensures a non-self-intersecting mesh, which result should the displacement occur in the tangential direction [4]. Most deformable model algorithms base the external force on the image gradient [8]. In the original paper, the external force is defined as follows(2)

The gradient force is determined from a search within a certain radius in the neighbourhood of each vertex for a voxel with the highest gradient intensity. The edge force is calculated by searching for the voxel with the highest intensity in the direction of the normal line of each vertex.

Despite their many intrinsic advantages, deformable model algorithms suffer from three main limitations [9]: (1) they are sensitive to initialization problems, which necessitates that they begin their deformation in close vicinity to the final desired solution in order to avoid becoming trapped in local minima; (2) they are often unable to adapt to boundary concavities due to internal forces which keep the model smooth and minimize curvature; and (3) they are prone to self-intersection unless adequately constrained by internal and external forces.

We have modified the deformable model algorithm of Delingette by adding four components to the original model to adequately segment MRI scans of multiple embryos acquired within the same field of view. First, we have added a balloon force component to the definition of external forces. This allows us to start segmenting all embryos from identical spheres without having to worry about model getting trapped in close by local minima at stated in the above. Second, a collision force component was also added to the definition of external forces. Third, we have implemented the concept of a tube mask to exclude the plaster walls of the sample tube. The latter two concepts help solve the problem of touching embryos. Finally, instead of segmenting one embryo at a time, we have designed and implemented a deformable model program which segments up to *N *embryos simultaneously.

In the sections to follow, we explain the modifications to the original deformable model algorithm of Delingette. The solution proposed herein, while designed with a particular application in mind, should be generally applicable for using deformable models to segment multiple similar and potentially touching objects within one image.

#### 2.1.2 Modified Deformable Model with Balloon Force

Initialization is one of the most challenging issues with deformable models. The success of most deformable model segmentation algorithms is determined by how closely the edges of the initial model follow that of the final result. If the initial model is much smaller than the object which is being segmented then the model will get stuck in local minima resulting in unsuccessful segmentation. In our case, since we start with identical initial models for all embryos, there is a need for a method to allow for local inflation of all models until they lie within a region of the image where they are close to the boundary of the embryos. To solve the multiple embryo initialization problem we implemented a two step solution wherein the image is fist coarsely classified into embryo versus gel, and secondly a balloon force is added to the deformable model to drive it towards the correct boundary.

**Figure 2:**
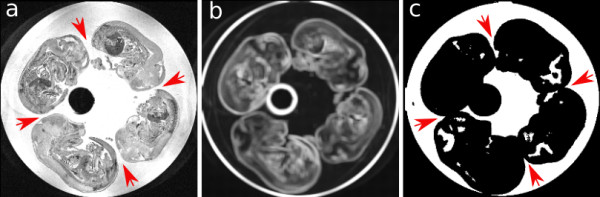
**Axial slice of image a) original, b) SD and c) classified image**. Note the arrows show the locations where the embryos are not touching in the original image but are intersecting in the classified image.

As mentioned in the previous section, due to non-uniform MRI intensity values across the gel and similar intensities between gel and embryo in the image, thresholding algorithms are unsuccessful at separating embryos from gel (Figure 2a). Therefore, there is a need for an algorithm which is capable of labeling each voxel in the image as embryo versus non-embryo. Here we employ a multispectral Bayesian supervised classification algorithm previously implemented to separate human brain images in grey matter, white matter, spinal fluid, and lesions [10]. The Bayesian algorithm is based on using a training set determined by the user to initialize the voxel classification - either based on a probability map or manually selected. The training set refers to a list of 3D coordinates voxel intensities for each class type in our case 25 points for class embryo and another 25 points for class gel. The Bayesian algorithm also uses statistical measures such as standard deviation (SD) from the data to further aid the classification of voxels in the image (Figure 2b). The algorithm then estimates the probability density function on the basis of centroid and covariance matrix evaluated for each class which is defined in the training set. Thus given an unknown sample, it is assigned to a class if a posteriori probability is maximized over the Bayes' theorem. To create our classified image, we first create a training set from the image with labels embryo or gel. We then use this set of points to calculate the probability density function for each of the two classes and further determine the maximum likelihood of any sample point belonging to each of our classes on the basis of Bayesian theorem. This will allow us to classify all voxels in image as explained above (Figure 2c).

It is important to mention that although the classification algorithm is capable of labeling the image with sufficient accuracy for initializing the deformable models, it has a tendency to label a few more voxels belonging to embryos than the actual embryos. As the problem of touching embryos already exist in the original MR images and is addressed by our algorithm, this does not create a problem for our technique.

**Figure 3:**
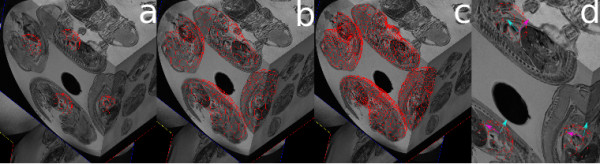
**Overlay of sphere simplex meshes and embryos**. a) initial simplex spheres overlaid with their corresponding embryos b) simplex meshes after 50 iterations c) refined simplex meshes after 50 iterations and the completion of segmentation and d) a close-up view with forces indicated, cyan for normal and purple for tangent forces. For simplicity only four deformable simplex meshes are shown.

Once the image is classified into embryo versus gel, a balloon force was designed to drive the mesh towards boundaries of the embryo while avoiding bad local minima using the classified image as a guide. It allows for the expansion of the initial spherical models until the edges of the model get close (2-4 voxels) to the edges of the embryos. Once the model edges are close to the embryo edges, the original deformable model strategy can be used to recover the edges of the embryos. We modify the external force of equation 2 as follows(3)

where the balloon force is defined as(4)

where the **B**(*I*) is the binary threshold operator on the classified image and **n **is the normal force at each vertex (Figure 3d).(5)

where *T_low _*and *T_high _*are the lower and upper thresholds for desired intensity values of the objects of interest and in our case were chosen as *T_low _*= - 0.99 and *T_high _*= 1.0. Together, the balloon force and the classified image expand the initial models towards the correct boundaries of the embryos; this alone is, however, not sufficient for the final segmentation results. As explained before, the embryos are larger in the classified image than their actual size and without the gradient forces to stop the expansion of models at the correct embryo edges, the balloon force expands the models past the edges of embryos and segments the gel right around embryos as part of the embryos. Further, a look at the classified image reveals that even if the embryos are not touching each other in the original image, they do intersect in the classified image, and hence without any intervention models will leak into each other as there are no barriers in between the embryos.

#### 2.1.3 Modified Deformable Model with Collision Force

To solve the problem of segmenting multiple embryos touching each other, there is a need for a collision detection algorithm. We have designed a simple collision detection algorithm which allows us to check for collision after every few stages of the deformation. Our approach is different than the traditional collision detection algorithms mainly because it checks the binary masks created from meshes using a simple rasterization algorithm instead of checking the meshes themselves [11]. In our algorithm, every mesh will have a unique collision energy image **C **hence **N **collision energy images for **N **meshes. Each collision energy image is created by "xor"ing all binary masks **M **except the one being deformed. For example, to create **C**_0_, we "xor" all binary masks **M **except **M**_0_. We create **N **collision energy images **C**_0 _to **C***_N _*for **N **meshes as follows

The collision energy image for each mesh can then be used to direct the deformation for that mesh away from all other meshes, hence creating collision detection. We have added another component to the external forces of equation 3 as follows(6)

where the collision force is defined as(7)

where the collision image acts as a binary thresholding operator for the image intensity **I**(8)

After every 5 iterations we check the vertices of each mesh for points of collision. We have picked 5 since it allows the meshes to deform but not so much that there is a chance of collision before detection. At each collision point a small neighbourhood of the voxels (5 voxels in our case, we have tested our algorithm with different neighbourhood of the voxels and determined that values greater than 5 do not add more efficiency but result in poor performance) are searched for the voxel with the highest gradient intensity hence **F***_gradient_*. Our collision energy is designed based on the assumption that the true boundary of the embryos is close by where the collision takes place between two meshes since they are expanding simultaneously. Therefore, a gradient search around the neighbourhood of the collision point reveals the correct boundary of the embryos.

The modified deformable model algorithm together with our collision detection method goes through the following steps to start deformation from identical spherical meshes and segment the embryos while avoiding collision between adjacent ones.

1. Use seed points determined by the user as the centers of identical spherical simplex meshes to initialize all embryos (Figure 3a).

2. Simultaneously inflate all meshes on the classified image to avoid any local minima using the balloon force. This will help the models get close to the boundary of interest, in our case the edges of the embryos (Figure 3b).

3. While expanding the models, use the collision detection algorithm after every 5 iterations to detect any collision between the models. If collision occurs, stop the inflation for models in collision and continue inflation for the rest of the models. Continue until all models have stopped inflating.

4. At this point, all models are within the vicinity of their corresponding embryos, use the gradient search algorithm to find close by edges of embryos (Figure 3c).

5. Once the deformation have stopped for all the models, use a rasterization algorithm to create a binary mask for each embryo from the corresponding simplex deformable model.

#### 2.1.4 Modified Deformable Model with Tube Mask

**Figure 4:**
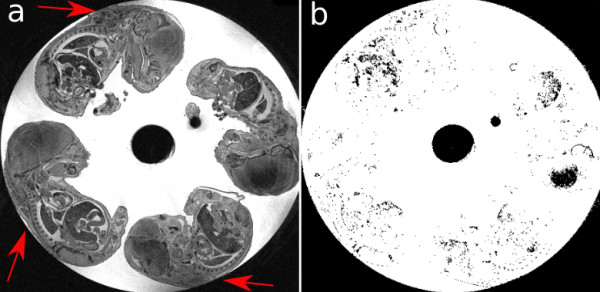
**Original image with arrows a) showing embryos touching the tube b) with corresponding slice of the tube mask**.

**Figure 5:**
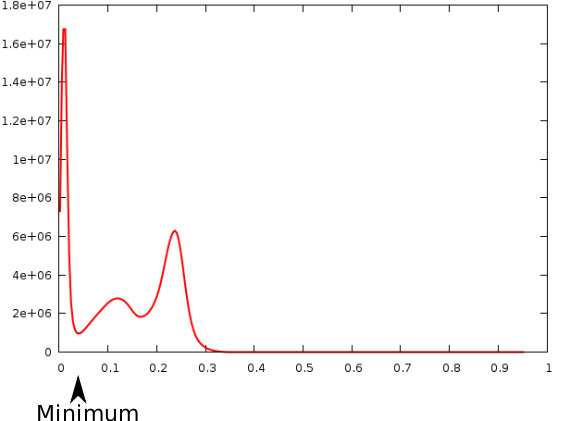
**A typical histogram created from one of the embryo images**. Note the arrow shows the minimum intensity value.

The collision image energy described in the previous section is capable of taking care of embryos touching each other but is unable to deal with cases where embryos are touching the walls of the sample tube (Figure 4a). In these cases, there is no clear distinction between the embryo boundary and the edges of the tube, hence making it difficult for the deformable model to stop at the correct edge. To ensure that models will not leak into the outside of the tube we need to create a mask for the entire tube in the image and ensure the vertices of the models do not cross beyond the tube mask boundary. Thus there is a need for an algorithm which is capable of labeling the image to background versus the rest of the image (i.e., tube) in our case. We adapted the BET brain segmentation algorithm [12] for this purpose. This algorithm uses the intensity histogram of the image to find a robust lower and upper intensity values, while ignoring small number of voxels which have widely different values, to label brain versus background voxels as the basis of its segmentation. In our case, designed for labeling tube versus background voxels, a tube mask image is created from the original image. The lower intensity is found by calculating the mean value of the bin which holds the local minimum after the first highest peak (Figure 5). The maximum intensity is simply taken from the data range of the image. Using these two values, thresholds are calculated which attempt to distinguish between the tube and background in the image. A binary threshold filter is then applied to the original image with the calculated high and low threshold values to create the final tube mask (Figure 4b).

At each iteration the final displacement of the model points are created and checked to make sure the displacement lands every point within the tube mask. If it happens that any point is displaced outside the tube, the displacement for that point is set to zero and the previous coordinate is assigned to the point ensuring that no point leaves the tube mask area.

We have modified the original definition of the deformable simplex model algorithm with balloon force, collision force and tube mask to simultaneously deform up to *N *models while ensuring that they do not leak into each other or outside of the tube.

#### 2.1.5 Multi-resolution Approach

The gradient forces which attract the model towards the correct boundaries of the target are computed around each model point within a local region in the image and often gets the model stuck to spurious image features or non-true boundaries of the object. Therefore, deformable models must be initialized close to the boundaries of the object to avoid unsuccessful segmentation. This feature of deformable model algorithms is also present in our modified version of the deformable model as it too searches the image within a local region of model points and can get the model stuck to non-true boundaries of the object.

The multi-resolution approach allows the deformable model to pass over non-true boundaries of the object and to quickly find a rough boundary approximation in the early stages of deformation [9].

Another important reason for starting the deformation with low resolution models is that because our deformable model algorithm does not have any explicit checking for self-intersection, starting the deformation with high resolution models will likely result in self-intersecting models due to having too many points at the first stage of deformation at which the models are expanding by the balloon force. Self-intersecting models result in binary masks with holes in them and since our collision detection algorithm relies on the binary masks created from the models, having non self-intersecting models is essential to the success of our algorithm.

Our deformable model algorithm starts with low resolution models. After the first stage of the deformation, the models go through major expansions with minimal or no self-intersection. However, low resolution models only recover approximate edges of the target boundaries. To recover finer boundary features of the embryos high resolution models are needed. We increase the resolution of the models by a two fold after every stage of the deformation and continue until achieving satisfactory results and the algorithm has reached the convergence criteria.

### 2.2 Implementation

Initialization is one of the main problems with most deformable model algorithms. The deformable model algorithms usually can only find the close by edges once the model is placed within the close vicinity of the shape of interest. Although we use the balloon force (section 2.1.2) to locally inflate the initial meshes and get close to the boundaries of interest, we still need a starting point for each individual mesh. Thus, there is a need for a separate method to determine the initial location of the models before segmentation can take place [5]. Delingette has proposed automatic methods for initialization, however, these algorithms have very limited range of application and are sensitive to image noise and most importantly are not designed for multiple object initialization which is required in our case since we are deforming multiple models simultaneously [4].

**Figure 6:**
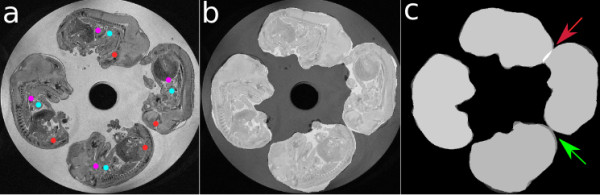
**Sensitivity of seed initialization**. a) original image with seed locations set 1 (cyan), set 2 (purple) and set 3(red) b) original image overlaid with masks created using set 1 of seeds c) overlaid of masks created using set 1 and set 2 of seed locations for segmentation. Set 3 of seed locations are too close to the embryo edges and result in unsuccessful segmentation. The green arrow shows the area of difference between two masks. The red arrow shows the area where the two masks intersect.

Our deformable model algorithm uses manual initialization. A user must determine *N *seed points from the image before attempting to segment *N *embryos for each dataset. We define the seed point as any 3D point which lies within the boundaries of an embryo, preferably in the centre. The seed points are then used as the centres of *N *equal size spherical simplex meshes. Once the seeds are determined and the meshes are initialized, the algorithm starts deformation. The location of a seed point for initialization of any deformable model algorithm is defined as a point within the boundaries close to the center of the object of segmentation. In our case, we have tested our algorithm with seed points within 10 voxels of each other and had successful results while keeping all parameters identical (Figure 6a, cyan and purple points). However, seed points which are very close to the embryo edges resulted in unsuccessful segmentation (Figure 6a, red points). An overlaid view of masks created for segmenting the embryos with set 1 of seed points of 4 embryos is shown in (Figure 6b). These masks are also overlaid with masks created from segmenting the embryos using set 2 of seed points (Figure 6c). To determine the sensitivity of our algorithm to initialization, we have segmented one set of 20 embryos with three different sets of seed points and determined the mean and standard deviation of the overlapped masks to be 0.94 ± 0.08.

**Figure 7:**
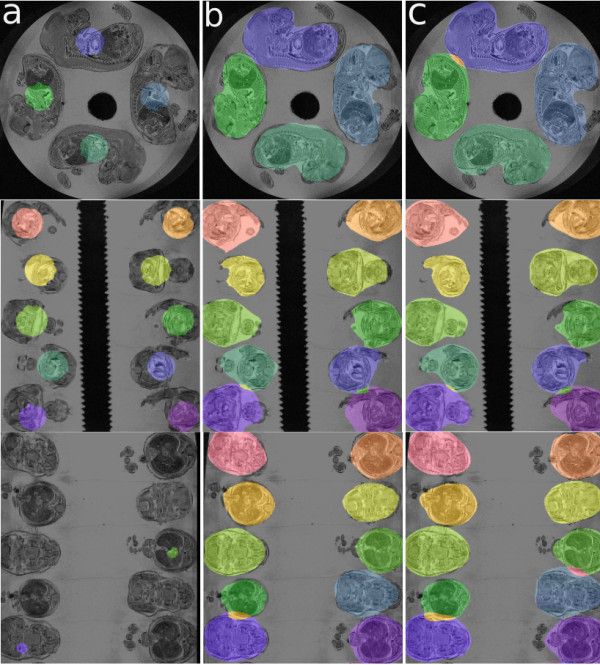
**Different views of embryos with initial mask column a**. Each colour shows a different mesh separate from other meshes. Due to small sizes of the initial meshes, they do not show in all views of the image. Similar views of embryos are shown after the first stage of deformation (column b), and finally after the last stage of deformation (column c). The small areas between adjacent embryos showing in different colour than the embryos are shared by both embryos. Note, low resolution meshes in early stages of deformation with rough edges versus smooth edges of high resolution meshes in final stage of deformation.

Our Algorithm is designed for simultaneous multiple object segmentation. This means that once the seed points are determined by the user, the algorithm initializes as many identical spherical deformable simplex meshes as the number of seed points and start the deformation on all initial models simultaneously. Four initial models are shown as masks (binary images) overlaid on their corresponding embryos (Figure 7: column a).

All forces that are governing our algorithm are strengthened or weakened through the use of weight factors (i.e., parameters). Every parameter ranges from zero (no strength) to one (highest strength). We have determined the particular values for each of our parameters through experimentation. At the start of the deformation, edge forces are kept at zero as it is not being used. Balloon forces and internal forces are kept high at 0.08 and 0.9 respectively, this is to ensure that the meshes are expanding rapidly while being kept smooth and hence free of self-intersection. The gradient and collision forces are kept at a middle range at 0.3 and 0.4 respectively to make sure mesh expansion is possible to the maximum limit but not beyond the embryo boundaries. Simplex meshes also benefit from having control over the scale of smoothness which is defined as the size of the neighborhood around each point used for smoothing of the mesh. To avoid self-intersection to the maximum level, the scale of the smoothness is also kept very high at 12 at the early stages of deformation.

After the first stage of deformation, the meshes are expanded dramatically and are close to the true edges of the embryos (Figure 7: column b). At this stage, the resolution of the mesh is increased by two fold, balloon and internal forces are decreased to 0.02 and 0.7 respectively. The balloon force is decreased because there is no need for further expansion of the meshes as they are already close to the edges of the embryos. The internal forces which keep the meshes smooth is also decreased so as to allow the meshes to recover finer edges of the embryos. The gradient force is increased to 0.4 to make sure the meshes are attracted to the edges of the embryos and the collision force is decreased to 0.3 to avoid collision between touching embryos while further deformation is still taking place. The smoothness scale is also decreased to 3, since self-intersecting meshes mostly occur during the first stage of deformation when meshes go through major expansion.

It has been observed that if the collision detection force is kept at a very high level of 0.8-0.9 to keep the segmentation of touching neighbouring embryos free of any intersection, the embryos will not be fully segmented. To overcome this problem, the collision detection force is reduced to mid range of 0.3-0.4 to allow slight touching of embryos so they can be fully segmented.

The stage of deformation with high resolution models can take place between 3 to 5 times in order to recover all fine feature of the embryos or when convergence is reached (Figure 7: column c). However, convergence does not imply that the modified deformable model algorithm is capable of recovering fine features such as paws or tails of the embryos. To recover such fine features using the simplex model deformable algorithm either the features have to be present in the initial model (i.e., to recover the tail, there has to be a tail present in the initial model) or there is a need for an algorithm which is capable of refining the model locally i.e., increase the resolution of the mesh only around areas with fine features.

The deformation continues until the models are stabilized. To determine the stability of each mesh, each point of the simplex mesh is classified as "active" or "inactive". A point is considered "inactive" if the magnitude of its displacement (the difference between its old position versus its new position) is less than a user defined threshold of 0.0001 in our case. The activity of each simplex mesh during the last **n **iterations is defined as the ratio of its inactive points over the total number of points. Any mesh with activity ratio of higher than a user determined threshold value of 0.5 is considered stabilized hence converged [9].

**Figure 8:**
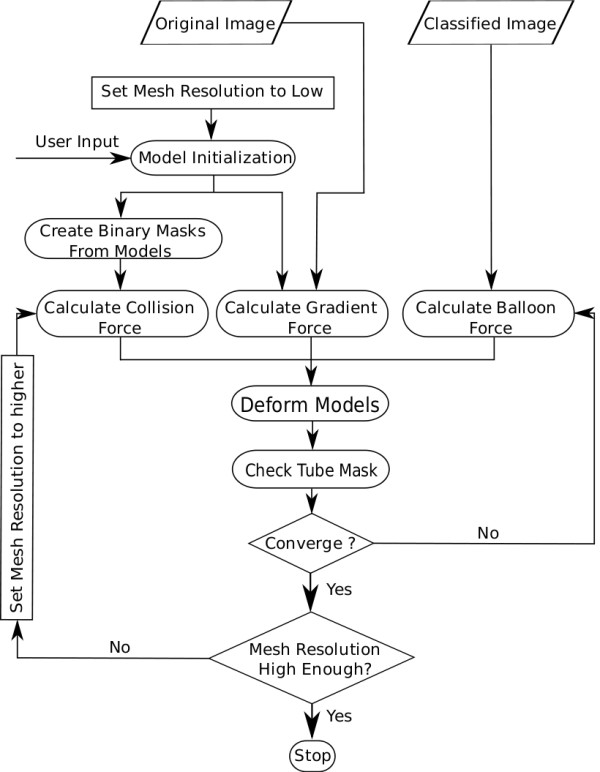
**Multiple-embryo multi-resolution segmentation**. The deformation starts with low resolution meshes with user input as centers of the meshes. As the deformation proceeds and meshes get closer to embryo boundaries, the resolution of the meshes is increased so finer edges can be recovered.

The deformation continues for meshes which are not converged while disabling the meshes which are converged until all the meshes are stabilized. Figure 8 gives an overview of our modified deformable model algorithm with collision detection.

## 3 Results and Discussion

**Figure 9:**
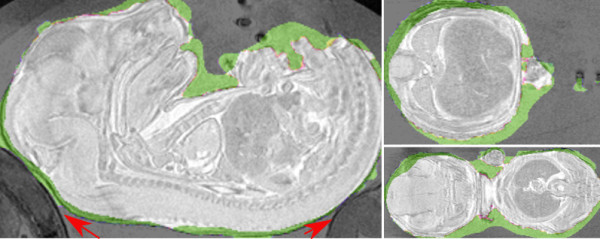
**Different views of one embryo overlaid with masks manual (green) and automatic (white) segmentation masks with the intersection of the two masks shown in white**. Note the red arrows show the locations of the other touching embryos.

Using identical parameters, we have segmented 3D images of six tubes of multiple embryos provided by Oxford university. Figure 7 shows the results of the segmentation at different stages of the deformation of one of the datasets. Figure 9 shows the closeup view of one embryo with manual and automatic segmentation.

**Figure 10:**
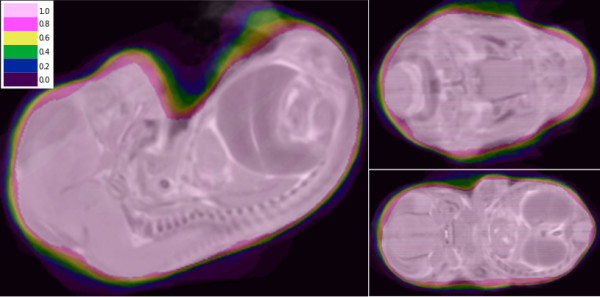
**Overlaid views of the average embryo masks of 20 registered embryo masks (our algorithm) with an average registered embryo of the same 20 embryos segmented manually**. As expected, all our masks cover the head and body of embryos with maximum alignment, all masks cover the area labeled 1.0. Alignment decreases as we go through the various embryo masks for instance label 0.4 shows all masks which cover head and body and part of paws (an area that our algorithm is not capable of segmenting).

We validated the deformable model algorithm against manual segmentations of 37 embryos originating from two tubes of 20 and 17 embryos respectively. Examples of manual and automated segmentation are shown in Figure 9. A quantitative summary is shown in Table 1. In order to assess the source of error in the deformable model algorithm, we used non-linear image registration to bring all embryos and their corresponding segmentations into alignment [13]. As can be seen in Figure 10, the maximum error occurs around the tail and paws of the embryos.

**Table 1:** Mean and standard deviation of kappa, specificity and accuracy for two tubes totaling 37 embryos.

Tube	Kappa	Specificity	Accuracy
Tube 1	0.83 ± 0.07	0.83 ± 0.04	0.88 ± 0.03
Tube 2	0.78 ± 0.06	0.87 ± 0.03	0.88 ± 0.03

Given that the primary error was in the tail and paws, we next checked whether organs were included in the automatically generated mask. To do this, all the organs were first manually segmented on the two average embryo images created in the previous section. Using the inverse transformations, the segmented organs were then registered to each individual mask created by our program and the results were compared. Table 2 reports the mean and standard deviation of four different organs for the two tubes. The liver, lung, and ventricle of the heart were fully included in the embryo masks in every single case. Minor errors were found in the brain.

**Table 2:** Mean and standard deviation of brain, liver, lung and ventricles for two tubes totalling 37 embryos.

Tube	Brain	Liver	Lung	Ventricle
Tube 1	0.998 ± 0.0	1 ± 0.0	1 ± 0.0	1 ± 0.0
Tube 2	0.991 ± 0.02	1 ± 0.0	1 ± 0.0	1 ± 0.0

## 4 Conclusions

We have developed a novel multi-resolution segmentation technique for semi-automatically segmenting multiple embryos simultaneously. Our multiple embryo segmentation technique can also be applied to other multiple object segmentation problems with possible touching of objects. Our algorithm was found to be highly accurate in capturing the whole embryo as well as its organs. Our deformation scheme follows a low to high resolution approach at which we start the deformation with low resolution meshes and continue increasing the resolution of the meshes through higher stages of deformation. The meshes are then deformed using a combination of internal and modified external forces (i.e., balloon and collision forces). The multi-resolution approach allows us to use identical initial models for all embryos while quickly and efficiently recovering the rough overall anatomical structure of the embryos at the first stage of deformation. The final shape of the embryo is then recovered by subsequently capturing the finer details of the embryos with higher resolution meshes at every stage of the deformation.

We have designed and implemented the concept of balloon force to allow us to start the deformation with small spheres and successfully reach the boundaries of the embryos. Using small initial models is a major issues with most deformable models as they can get trapped in local minima. However, our algorithm has overcome this problem by introducing the concept of balloon force.

To solve the problem of touching embryos, a novel collision detection algorithm was introduced. The collision detection method allows us to segment multiple embryos while avoiding major leaks into adjacent neighboring embryos. To our knowledge, most collision detection algorithms work on triangle meshes only whereas in our case binary masks created from the meshes are used. In order to recover the complete shape of all embryos, collision forces were kept in such a way that a small collision between the embryos was allowed.

Our multi-resolution deformable model algorithm with collision detection and balloon forces has overcome some of the difficulties of deformable model algorithms while allowing us to segment multiple embryos simultaneously. It has also enabled us to segment as many embryos without having to worry about the touching embryos. Our algorithm is written entirely in C++ as part of The Insight Toolkit(ITK) itk.org-InsightApplications/DeformableModelSimplexMesh open source software. It takes about half an hour to make the initial preparations including determining the seed points, creating the classified image and creating a single text file which holds all the information such as the location of the images and all parameters for the deformable model application. After the initial preparation, it takes approximately 6 hours to complete the deformation of 32 initial spheres, creating 32 individual masks on a 64-bit PC with 3600 MHz Intel Xeon CPU where each tube dataset is about 0.5 GB in size with 50 × 50 × 50 *μ*m resolution. This is computer time in comparison to segmenting embryos manually on high resolution datasets which are labour intensive with roughly 10 hours of operator time for segmenting one embryo.

While we were unable to find alternate solutions to the problem of segmenting multiple similar and touching objects in the literature, we acknowledge that there are other advanced segmentation methods, such as level sets, graph cuts, or similar region growing algorithms, that could have been used instead. The most likely alternate solution, level sets, involve numerical methods for tracking the evolution of contours and surfaces which uses image-based features such as mean, gradient and edges in the governing differential equations to segment the image [14]. The same issues of preprocessing that we addressed with the pro-posed deformable model approach would likely have to be solved for a level-sets based implementation.

Although our multi-resolution deformable model algorithm is capable of successful segmentation of multiple embryos while avoiding major leaks into neigh-boring embryos, it is still not considered fully automatic as it requires user's input for seed points at the beginning. We would like to explore methods at which the initialization of multiple seed points can be done automatically and without any user intervention. Further, our algorithm does not use any methods to avoid self-intersecting meshes during the deformation. Currently, we have solved this issue by carefully choosing a high scale of the deformation at the first stage of deformation. Although successful most of the time, this stage of our algorithm is rather slow comparing to other stages of the deformation. For this reason, we would like to investigate algorithms which can be added to our program for automatically avoiding self-intersecting meshes. Finally, we would like to be able to recover finer details of embryos by using a local refinement technique. This implies that the meshes are not consistently refined everywhere but are locally refined at places of high curvature similar to -subdivision algorithm [15].

## Competing interests

The authors declare that they have no competing interests.

## Authors' contributions

LB has modified the original deformable simplex mesh algorithm with different forces specifically the collision force to create simultaneous multiple mouse embryo segmentation. MZ has manually segmented 37 embryos and registered them together, her results is used to validate the results of the automatic segmentation. JES and SB have designed and implemented the protocol for multiple mouse embryo MR imaging and provided the images used in this paper. JGS, RMH and JPL assisted with the design and development of the algorithm and the drafting of the manuscript. All authors read and approved the final version of the manuscript.

## References

[B1] Nieman Brian J Bishop Jonathan Dazai Jun Bock Nicholas A Lerch Jason P Feintuch Akiva Josette Chen X Sled John G Mark Henkelman R MR technology for biological studies in mice Nuclear Magnetic Resonance in Biomedicine 2007 20 291 303 10.1002/nbm.1142 17451169

[B2] Bock Nicholas A Konyer Norman B Mark Henkelman R Multiple mouse MRI Magnetic Resonance in Medicine 2003 49 1 158 167 10.1002/mrm.10326 12509832

[B3] Schneider Jürgen E Böse Jens Bamforth Simon D Gruber Achim D Broadbent Carol Clarke Kieran Neubauer Stefan Lengeling Andreas Bhattacharya Shoumo Identification of cardiac malformation in mice lacking ptdrs using a novel high-throughput magnetic resonance imaging technique BMC Developmental Biology 2004 16 4 10.1186/1471-213X-4-16 PMC545075 15615595

[B4] Delingette Hervé General object reconstruction based on simplex meshes Int Journal of Computer Vision 1999 32 111 142 10.1023/A:1008157432188

[B5] Delingette Hervé Simplex meshes: a general representation for 3D shape reconstruction Proc of Int Conf on Computer Vision and Pattern Recognition (CVPR'94), Seattle, USA 1995 856 860

[B6] Galdames Francisco Jaillet Fabrice From Triangulation to Simplex Mesh: a Simple and Efficient Transformation Technical Report RRLIRIS-2010-021, LIRIS UMR 5205 CNRS/INSA de Lyon/Universit Claude Bernard Lyon 1/Universit Lumire Lyon 2/cole Centrale de Lyon 2010

[B7] Tejos Cristian Irarrazaval Pablo Crdenas-Blanco Arturo Simplex mesh diffusion snakes: Integrating 2d and 3d deformable moels and statistical shape knowledge in a variational framework International Journal of Computer Vision 2009 85 1

[B8] McInerney Tim Terzopoulos Demetri Deformable models in medical image analysis: A survey Medical Image Analysis 1996 1 2 91 108 10.1016/S1361-8415(96)80007-7 9873923

[B9] Park Joo-Young McInerney Tim Terzopoulos Demetri Kim Myoung-Hee A non-self-intersecting adaptive deformable surface for boundary extraction from volumetric images Graphics and Computers 2001 25 421 440 10.1016/S0097-8493(01)00066-8

[B10] Zijdenbos Alex P Forghani Reza Evans Alan C Automatic pipeline analysis of 3D MRI data for clinical trials: application to multiple sclerosis IEEE Transactions on Medical Imaging 1999 21 10 1280 1291 10.1109/TMI.2002.806283 12585710

[B11] Curtis Sean Tamstorf Rasmus Manocha Dinesh Fast collision detection for deformable models using representative triangles Proceedings of the 2008 Symposium on Interactive 3D graphics and games 2008 61 69

[B12] Smith Stephen M Fast robust automated brain extraction Human Brain Mapping 2002 17 3 143 155 10.1002/hbm.10062 12391568 PMC6871816

[B13] Zamyadi Mojdeh Baghdadi Leila Lerch Jason P Bhattacharya Shoumo Schneider Jurgen E Henkelman R Mark Sled John G Mouse embryonic phenotyping by morphometric analysis of MR images Physiological Genomics 2010 42A 2 89 95 10.1152/physiolgenomics.00091.2010 20682847 PMC2957795

[B14] Malladi Ravikanth Sethian James A Vemuri Baba C Vemuri. Shape modeling with front propagation: A level set approach IEEE Transactions on Pattern Analysis and Machine Intelligence 1995 17 2 158 174 10.1109/34.368173

[B15] Kobbelt Leif-subdivision Proceedings of SIGGRAPH 2000 Anual Conference on Computer Graphics, New Orleans, LA 2000 103 112

